# Role of bioactive compounds in the treatment of hepatitis: A review

**DOI:** 10.3389/fphar.2022.1051751

**Published:** 2022-12-21

**Authors:** Arpita Roy, Madhura Roy, Amel Gacem, Shreeja Datta, Md. Zeyaullah, Khursheed Muzammil, Thoraya A. Farghaly, Magda H. Abdellattif, Krishna Kumar Yadav, Jesus Simal-Gandara

**Affiliations:** ^1^ Department of Biotechnology, School of Engineering and Technology, Sharda University, Greater Noida, India; ^2^ Centre for Translational and Clinical Research, School of Chemical and Life Sciences, Jamia Hamdard University, New Delhi, India; ^3^ Department of Physics, Faculty of Sciences, University 20 Août 1955, Skikda, Algeria; ^4^ Biotechnology Department, Delhi Technological University, Rohini, India; ^5^ Department of Basic Medical Science, College of Applied Medical Sciences, Khamis Mushait Campus, King Khalid University, Abha, Saudi Arabia; ^6^ Department of Public Health, College of Applied Medical Sciences, Khamis Mushait Campus, King Khalid University, Abha, Saudi Arabia; ^7^ Department of Chemistry, Faculty of Applied Science, Umm Al‐Qura University, Makkah, Saudi Arabia; ^8^ Department of Chemistry, College of Science, Taif University, Taif, Saudi Arabia; ^9^ Faculty of Science and Technology, Madhyanchal Professional University, Bhopal, India; ^10^ Nutrition and Bromatology Group, Analytical and Food Chemistry Department, Faculty of Science, Universidade de Vigo, Ourense, Spain

**Keywords:** bioactive compounds, hepatitis, infection, treatment strategies, clinical trials

## Abstract

Hepatitis causes liver infection leading to inflammation that is swelling of the liver. They are of various types and detrimental to human beings. Natural products have recently been used to develop antiviral drugs against severe viral infections like viral hepatitis. They are usually extracted from herbs or plants and animals. The naturally derived compounds have demonstrated significant antiviral effects against the hepatitis virus and they interfere with different stages of the life cycle of the virus, viral release, replication, and its host-specific interactions. Antiviral activities have been demonstrated by natural products such as phenylpropanoids, flavonoids, xanthones, anthraquinones, terpenoids, alkaloids, aromatics, etc., against hepatitis B and hepatitis C viruses. The recent studies conducted to understand the viral hepatitis life cycle, more effective naturally derived drugs are being produced with a promising future for the treatment of the infection. This review emphasizes the current strategies for treating hepatitis, their shortcomings, the properties of natural products and their numerous types, clinical trials, and future prospects as potential drugs.

## Introduction

**FIGURE 1:**
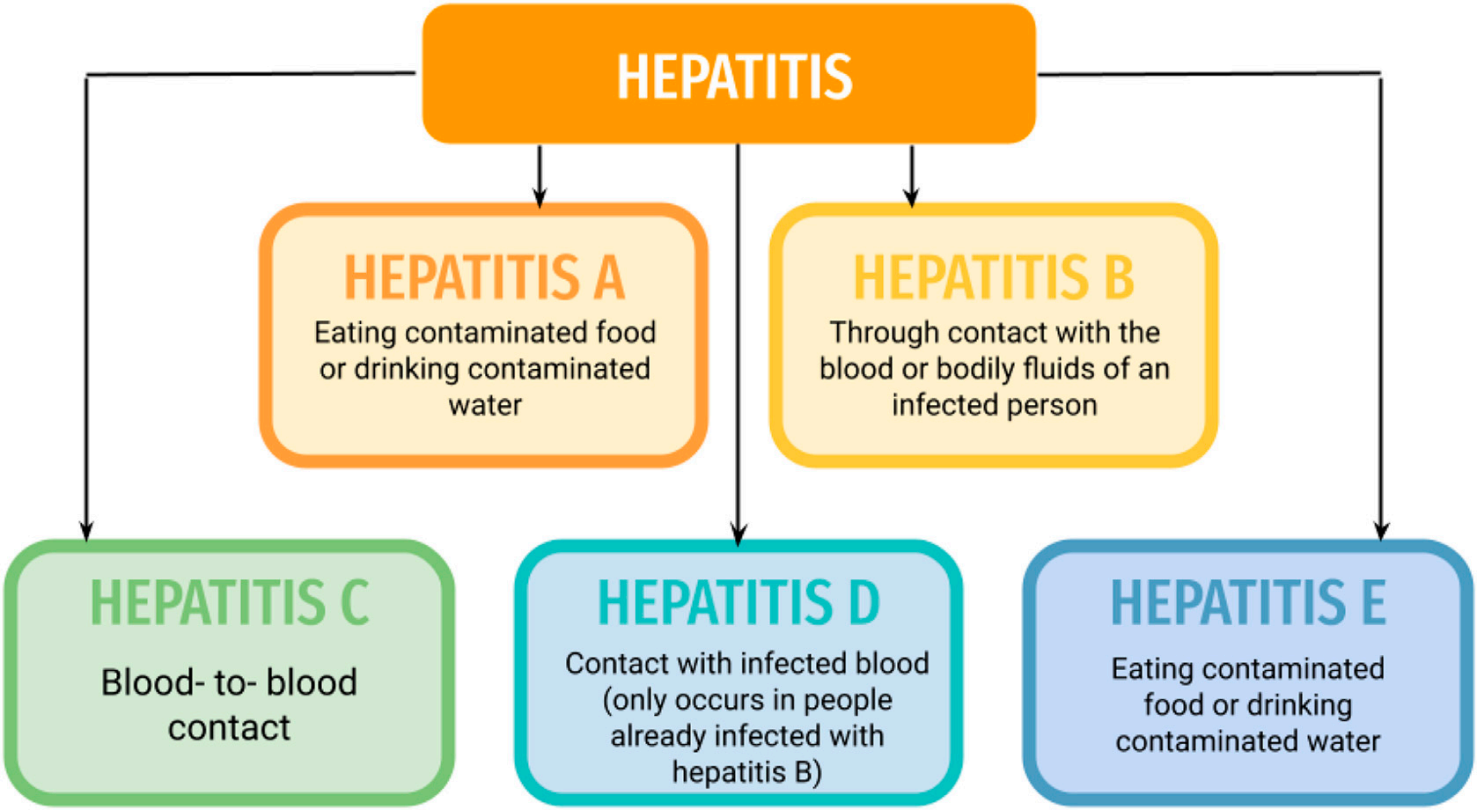
Transmission routes of Hepatitis types.

Hepatitis or as it is most commonly called, viral hepatitis is a severe/fatal disease. According to an estimated value, the complications of viral hepatitis approximately led to around 1–4 million deaths per year, throughout the world (Zarrin and Akhondi, 2021). Usually, various viruses could lead to inflammation of the liver like Epstein-Barr virus or Herpes simplex virus, or Cytomegalovirus, to name a few. But, all types of hepatitis viruses (A, B, C, D, and E), are causative agent references. Most hepatitis viruses lead to acute conditions and are self-limiting, but types B, C, and E can lead to chronic conditions. Chronic hepatitis can cause life-threatening conditions such as liver cirrhosis or hepatocellular carcinoma (González et al., 2017). Each year 1.5 million infections are reported due to Hepatitis-B and Hepatitis-C (Refer:https://www.who.int/news-room/fact-sheets/detail/hepatitis-b; https://www.who.int/news-room/fact-sheets/detail/hepatitis-c). Hepatitis viruses like A, C, D, and E are composed of RNA while the hepatitis-B virus is composed of DNA (Sinn et al., 2017; Shabanah et al., 2019; AlMalki et al., 2021). Hepatitis A virus (HAV) spreads through the contamination of food and water, in the feces of an individual. Hepatitis-B virus (HBV) exhibits vertical and distinctive transmission and can be transmitted through sexual passages (through secretions of vagina and semen), blood (through injections, drug abuse, etc) as well as by close human-to-human contact (MacLachlan and Cowie, 2015). Hepatitis C virus (HCV) spreads mainly *via* blood transfusions, but can also be transmitted *via* sexual contact, contaminated healthcare injections, and the use of drugs (like intravenous). Hepatitis-D virus (HDV) is transmitted through sexual or blood contact, like in the case of HBV and HCV. It is dependent on HBV as it needs HBsAg (HBV surface antigen) for its replication (Rizzetto, 2015). Hepatitis-E virus (HEV) spreads *via* contamination of food and water, and also *via* zoonotic route and transfusion (Figure 1).

Natural products have been shown to be extremely useful for curative and prophylaxis as well as palliative treatment of myriad conditions caused by bacteria, fungi, and viruses (AlMalki et al., 2021; Alghamdi et al., 2021; Shahid et al., 2021). Novel antiviral drugs have been designed and developed using natural compounds and modified into useful compounds for preventive and curative actions (Lin et al., 2014). Antiviral drugs that are derived from natural products are usually extracted from medicinal microbes, plants and herbs, animals, and humans. The most economically beneficial option for treatment involves the use of medicinal plants with minimum side effects (Roy and Datta, 2021). It has been seen that herbal drugs showed lower complications in comparison to chemical drugs, leading to few or no after-effects. In the last decade, the use of herbal products has increased because of the problems with chemical drugs. Additionally, herbal remedies show better efficiency in the case of some diseases, and for some others, only herbal remedies are present (Rabiei et al., 2015). Additionally, the currently available drugs have higher toxicity levels which pose the requirement of replacing these drugs with medications having a lower toxicity range (Zeinab and Kopaei, 2018). As a result, this review highlights the effects of different types of natural products in treating hepatitis, involving the current treatment options and the properties of natural compounds, and various studies proving their properties in the management of the disease, along with the future perspectives and clinical trials of the same.

## Hepatitis infection

HAV is a non-enveloped virus and belongs to the genus *Hepatovirus.* It can easily live outside the human body as well as in adverse conditions for a long time, which causes easy transmission of the virus and is the prime reason for past epidemics of jaundice. Its mode of transmission involves the fecal-oral route. HAV exhibits variable and nonspecific symptoms like jaundice, anorexia, fever, nausea, abdominal pain, fatigue, vomiting, fever, and nausea (Centre for Disease Control and Prevention, 2016). The severity of the HAV infection can be from asymptomatic to severe liver failure. Infants commonly suffer from asymptomatic viral infection, while adults experience symptoms such as jaundice.

HBV is a DNA (double-stranded) virus that belongs to the *Hepadnaviridae* family and is enveloped*.* The viral DNA is circular, partly double-stranded, and is the smallest known DNA virus. It exhibits replication in the hepatocytes and leads to liver dysfunction. The mode of transmission involves per mucosal or percutaneous route and blood transfusion, and also through vaginal fluid or semen to the uninfected person. The transmission of HBV can occur during childbirth (from mother to child), through sharing of needles, sexual intercourse, and transmission of blood through a mucosal surface or an open wound. HBV can lead to acute hepatitis with similar clinical manifestations as that of the Hepatitis A virus. But, the infection caused due to HBV is asymptomatic in 50% of the affected people (Centre for disease control and prevention, 2016). Less than 10% of the acute cases of jaundice prevail in the younger age group. HBV exhibits similar symptoms that HAV like nausea, fever, vomiting, abdominal pain, malaise, and flu-like symptoms (Centre for Disease Control and Prevention, 2016). The laboratory outcomes of HAV and HBV are identical as it demonstrates indistinguishable variations in the liver transaminases- ALT and AST are notably increased, during acute infection, sometimes greater than 1000 (Kim, 2009).

HCV is enveloped and transmitted through the blood of an infected person or through sexual transmission. It primarily affects the hepatocytes and if untreated/not treated properly, then it leads to liver cirrhosis in 20% of patients, which may cause hepatocellular carcinoma in later stages (Ringelhan et al., 2017). The immune systems (innate and adaptive) are evaded by HCV and chronic infections are caused in 70% of the patients (Ringelhan et al., 2017). Regular blood tests have significantly reduced transmission rates. Chronic HCV infection leads to severe conditions like liver cirrhosis, hepatocellular carcinoma, and fibrosis. Cryoglobulinemia vasculitis is one of the extrahepatic manifestations that is developed in about 2/3rd of the patients (Davuluri and Bansal, 2021). HCV replication can significantly affect the metabolism, which leads to inflammation and steatosis of the liver.

Hepatitis delta virus (HDV) belongs to the genus *Deltavirus.* The extracellular virions of HDV possess the single-stranded genomic RNA, covalently and circularly closed in a negative sense. It is a satellite virus and regulates packaging, release and transmission, depending on HBV (Netter et al., 2021). Acute viral infection can be caused either due to co-infection (when both HBV and HDV infection occurs simultaneously due to the same exposure) or superinfection (occurrence of HDV infection after the HBV infection like in the case of HBsAg positive patients). The clinical manifestation of the simultaneous infection corresponds to an acute infection caused due to HBV. Moreover, concomitant infection causes a high risk of acute hepatic failure. In addition to that, co-infection occurs in a biphasic manner in which two peaks of ALT levels are observed within a span of a short time, as HBV infection should occur first in order to begin the spread of HDV infection. Chronic HDV infection leads to higher levels of transaminases, especially in patients who are infected due to HBV. Chronic HDV infection causes serious liver disease with higher fibrosis progression rates as compared to HCV or HBV-affected patients.

The HEV is an RNA virus (positive sense) that is single-stranded. It is transmitted when the drinking water is contaminated by faeces or the meat of infected animals is consumed (Doceul et al., 2016) or also through iatrogenic transmission. The infection caused by HEV is asymptomatic or causes minor symptoms without affecting the liver in most individuals. Acute icteric hepatitis is a classic example of HEV infection that lasts for more than 2–6 weeks in about 5%–30% of patients. It includes a prodromal phase that leads to common manifestations such as fever, vomiting, body pain, malaise, and nausea and continues for up to 1 week. Jaundice and dark-coloured urine are primary signs of the icteric phase. In the convalescent phase, jaundice and other symptoms related to it resolve within a week or a few days. At the beginning of the prodromal phase and the initial icteric phase, the serum levels of alanine aminotransferase are significantly increased, and throughout the icteric phase, the levels of bilirubin are markedly high. About 0.5%–4% of patients affected with Hepatitis-E infection, suffer from acute liver failure (Blasco-Perrin et al., 2015).

## Current treatment strategies and their limitations

The treatment of HAV involves supportive management and prevention of the infection. Hepatitis virus infection can be effectively prevented by vaccination (Nelson et al., 2018; Centre for Disease Control and Prevention, 2020). At present, there are two single-antigen inactivated vaccines that are marketed in the United States- Vaqta, and Havrix. However, Vaqta demonstrates certain adverse effects like nausea, abdominal pain, appetite loss, diarrhea, joint pain, sore throat, etc (Refer: https://www.rxlist.com/vaqta-drug.htm#description).

Also, Havrix has also displayed some adverse events like complex regional pain syndrome, impaired work ability, oral discomfort, paresis, pelvic pain, etc. Twinrix is a single inactivated combination of both the vaccines Vaqta and Havrix, that is licensed for use (Centre for Disease Control and Prevention, 2019). However, it also has shown adverse effects like facial paresis, hypoesthesia facial, impaired driving ability, impaired work ability, monoparesis, paraparesis, paresis, pelvic pain, etc. Besides, nine approved drugs are available which can be used for treating chronic HBV, which include, two formulations of IFN (interferons)- one is conventional and the other is pegylated interferon, and there are 7 NAs (Nucleot(s) ide analogues)- tenofovir alafenamide fumarate, lamivudine, tenofovir disoproxil fumarate, telbivudine, entecavir, adefovir and besifovir dipivoxil (available only in Korea). The main objectives of therapy involve-the restriction of the advancement of the disease and upscale the rates of survival. PEG-IFN (pegylated-interferon) alpha along with ribavirin was conventionally used for 3–4 months for the therapeutic care of HCV infections (Ray et al., 2015). However, it showcased a broad spectrum of neuropsychiatric adverse effects like anorexia, depression, and sleep disturbances. PEG-IFN-α is also effective against HBV and HDV infection (Wedemeyer et al., 2011, Wedemeyer et al., 2019). But, its use can cause conditions like leukopenia and thrombocytopenia, which may lead to the discontinuation of the medication or modification in dose (Perrillo, 2009). Bulevirtide, which is an entry-inhibitor, has acquired conditional based approval from the European Medicines Agency (EMA) but the outcomes of the phase-3 trial are yet to be obtained. The phase-3 trials of Lonafarnib (prenylation inhibitor) are still ongoing. Additionally, other potential agents like RNA interference substances, nucleic acid polymers, and IFN-lambda are currently being examined. NA (Nucleot(s) ide analogues) exhibit inhibition of the reverse transcription of the HBV, but it does affect the replication of HDV. But, NA is effective in case of severe liver disease and against the viral DNA in case of co-infection with HBV and HDV (Lampertico et al., 2017). However, the disadvantages that are associated with nucleotide analogues (NAs) include the decreased rates of seroconversion of HBsAg and HBeAg and long-term therapy in most of the infected people (Zoulim and Durantel, 2015). Additionally, NAs do not affect the activity and level of cccDNA that exists in the liver of the infected person, even after the treatment with antivirals. Therefore, it takes time for NAs to exhibit their therapeutic actions and requires unspecified therapy.

## Natural products

In recent times, natural products have been shown to be of great use in the therapy of hepatitis, with less drug resistance and adverse effects (Cai and Qin, 2019; Xu et al., 2019). Consequently, more studies are being conducted to comprehend the actions of natural products (Duan and Chen, 2016; Ahmed et al., 2017; Yao et al., 2019). Some reports have proven that numerous natural medicines having novel structures as well as anti-HBV properties might be good drug candidates for hepatitis B infection. Although such studies were primarily included in the recognition of products displaying anti-viral effects against HBV; the mechanism and targets of the products were fewer. The mechanism of action of therapeutic drugs like NAs as well as interferons, on anti-Hepatitis B Virus, is evident, but the unfolding of the drug-resistant mutants of HBV usually reduces the therapeutic activities. Therefore, the production of safe and efficient anti-HBV medications having unconventional mechanisms is the main target of today’s research (Li Y. T. et al, 2012; Cai and Qin, 2019). Some of the different kinds of natural compounds having anti-HBV properties are flavonoids, phenylpropanoids, alkaloids, glycosides, terpenes, lactones as well as organic acids. Because of the wide popularity of natural products for treating and preventing diseases, recently, pharmaceutical companies have been developing new antimicrobial formulations which are derived from such compounds. For instance, phytotherapy or use of medicinal plants is practised all over the world, specifically in developed nations like some of the European nations as well as the United States (Solati et al., 2017). Around 45% of the marketed natural products that are utilized for the management of infections caused by the hepatitis virus are obtained from medicinal extracts of plants or their derivatives (Lahlou, 2013). In addition to this, a global upsurge in the isolation of active compounds from medicinal plants had emerged in health care.

The hunt for new bioactive compounds is still prevalent in prime therapeutic areas like immunosuppression, metabolic and infectious diseases, as well as oncology. It has been an enormously studied part of pharmaceutical research for many years (Newman and Cragg, 2012). Around 40 new drugs have been launched between the time period of 2000- 2010, derived from plants, microorganisms, marine organisms, and a few chordates (Brahmachari, 2011). Furthermore, the WHO estimated that around 80% of the global population depends on conventional medications, mostly derived from plants, for their primary healthcare. The active compounds originating from herbs or plants are utilized either for therapeutic treatments or are administered through the oral route to infected patients in the form of powders, teas as well as other herbal formulations. Further, even phenolic products cause the bioactivity of the unrefined extracts of plants. In recent decades, researchers have attempted to recognize the bioactive compounds of these traditional medicines by systematically screening the natural products obtained from the extracts of herbs or plants and then testing their effectiveness using appropriate assays (on the basis of the studied pathology). One of the primary benefits of natural compounds extracted from plants is the lower manufacturing costs, due to the absence of the requirement for chemical synthesis. Such production leads to lesser expensive treatments and is available for low-income populations. Apart from this, different natural compounds have proven to exhibit antiviral effects against the influenza virus, HIV, herpes simplex virus, influenza virus as well as HBV and HCV. Further, the screening and development of natural compounds have resulted in the detection of effective inhibitors that inhibits the growth of the virus. However, there are various limitations as well associated with natural products. For instance, the extraction process of natural products from organisms is a cumbersome task. Along with this, the mode of extraction process is dependent on the type of compound to be extracted. To increase the varieties of the extracted natural products, the bioactive compound can be extracted in the presence of several solvents of varying polarity. Apart from this, another limitation is to identify novel natural products as some of the potential source organisms are difficult to be produced or culture in the laboratories as they only survive in their ecosystem. Such challenges are now being tackled by establishing novel methodologies for culturing for natural product synthesis induction. Therefore, naturally derived compounds of different origins have been shown to be of therapeutic use for hepatitis infection. The detailed studies of numerous natural products have been explained in the following sections, with specific emphasis on their role in inhibiting viral infection.

## List of natural products

**FIGURE 2:**
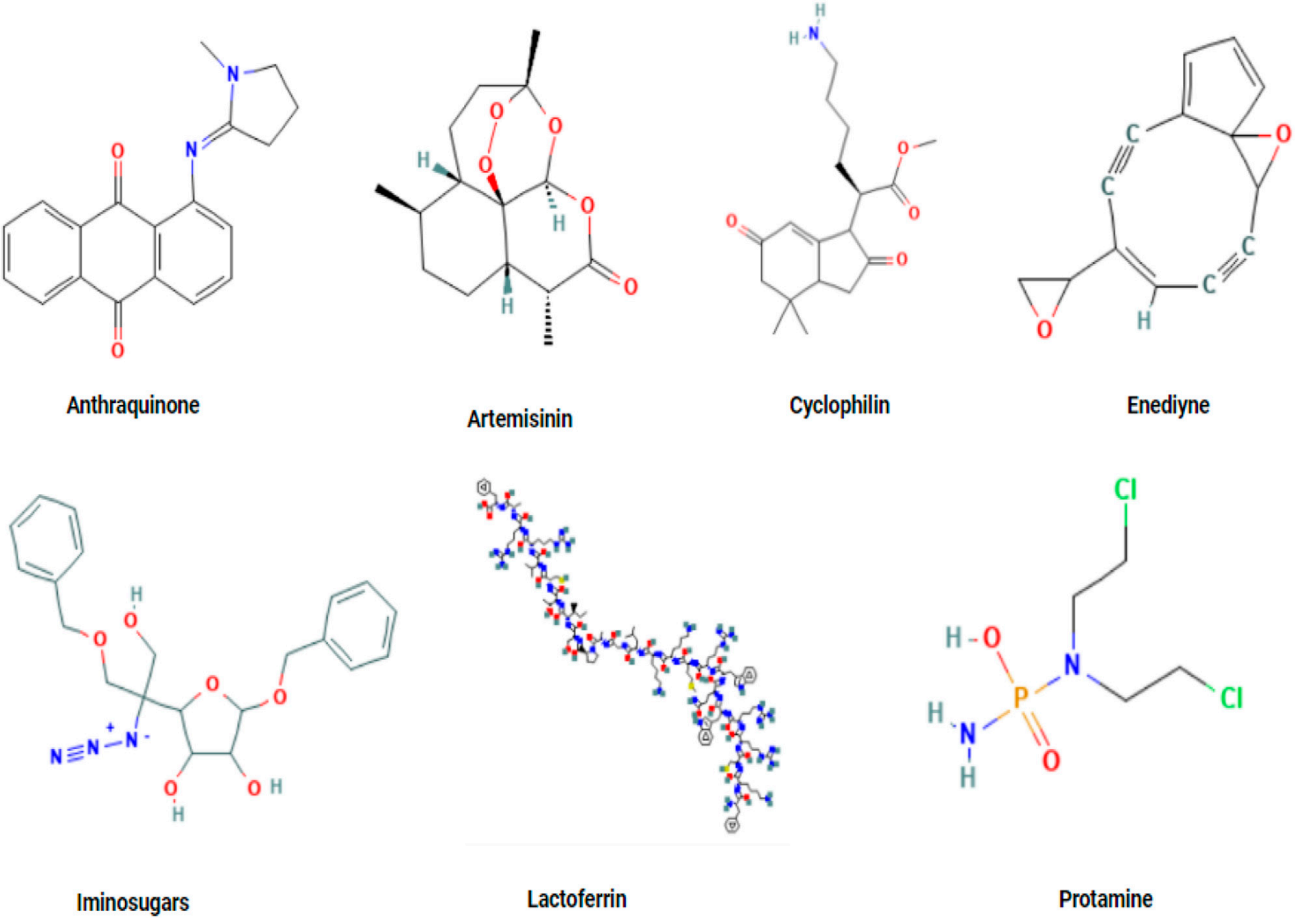
(I) HBV life cycle: (II) HCV life cycle.

Natural products have the potential treat various diseases one of them is hepatitis. Various natural compounds have been studied for effective results against hepatitis (Figure 2).

### Alkaloids

Alkaloids are natural compounds that possess a complex ring and nitrogen heterocyclic structure, which is responsible for most of their physiological effects. They also exhibit anti-microbial, anti-inflammatory, anti-cancer, and antioxidant activities. Jiang et al., 2013, reported that ethanol extract which was derived from the fruit of *Piper longum* L. fruit exhibited effective antiviral activity, and its specific derivatives demonstrated significant activities against the production of HBeAg and HBsAg on the cell line of HepG 2.2.15. This study also demonstrated that one of such compounds demonstrated the inhibition of the production of HBeAg and HBsAg at IC_50_ values of 0.21 and 1.80 mM. One more experiment by Zeng et al., 2013 exhibited that one of the alkaloids, namely DHCH, extracted from the *Corydalis saxicola plant,* caused effective inhibition of HBeAg and HBsAg production in HepG2.2.15 cells, along with TI of around 6.77 and 7.32. Furthermore, DHCH was shown to decrease DNA and cccDNA levels in time and dose-dependent ways, at IC_50_ values of 7.62, 8.25, and 15.08 μM.

### Anthraquinones

Anthraquinones are derived from the metabolites of fungi and lichens. They exhibit purgation, immunoregulatory, anti-cancer, and anti-inflammatory actions (Wang et al., 2019). Recent studies demonstrated the anti-HBV effect of anthraquinones (Bu et al., 2019). Sulochrin, (−)-2′ R-1-hydroxyisorhodoptilometrin, questinol, monochlorsulochrin, endo crocin, dihydrocodeine, asterric acid and (+)-2′ S-isorho-doptilometrin are extracted from the aciduric fungus *Penicillium* sp. Which is mangrove-derived, significantly inhibits the secretion of HBsAg than 3TC (positive control) at a specific dosage (Qin et al., 2016). Discovered that hypericin effectively decreased the viral DNA expression and also the HBeAg and HBsAg expression, like lamivudine (3 TC).

Another study by Peng et al. (1970) reported that anthraquinones, anthraquinone bile acid conjugates, and rubiadin exhibited activities against HBV infections on the cell line of HepG2.2.15 at IC_50_ of 12.41, 8.03, 17.05, and 8.13 g/ml. Rubiadin is a compound that not only effectively decreases the production level of HBsAg and HBeAg, inhibits the HBV DNA replication, as well as prevents the activities of the HBx protein and the growth of cells in a dose-dependent method, but may also result in an unconventional candidate of the anti-HBV agent. Parvez et al. (2019), first time demonstrated the anti-HBV property of anthraquinones which are AV-derived, probably through inhibition of HBV-DNA polymerase.

### Aromatics

The six phenols namely, ethyl 2,5-dihydroxybenzoate, m-hydroxybenzoic acid, ethyl 3,4-dihydroxy-benzoate, p-hydroxybenzoic acid, 3,4-dihydroxybenzoic acid, and m-hydroxy benzenmethanol. Aromatics demonstrate anti-microbial, anti-pyretic, analgesic, and anti-inflammatory properties. They demonstrated anti-Hepatitis B virus activities by causing inhibitory actions against the production of HBsAg as well as HBeAg at IC_50_ levels of 0.23–5.18 mM, and replication of HBV-DNA at IC_50_ of 0.06–2.62 mM. In addition to this, p-hydroxybenzoic acid, m-hydroxybenzoic acid, and m-hydroxy benzenmethanol exhibited anti-HBV actions at IC_50_ levels of 5.18, 3.76, and 4.55 mM against the secretion of HBsAg and 2.54, 2.36, and 2.62 mM for the inhibition replication of HBV-DNA (Cao et al., 2015). Zhou et al. (2014) demonstrated that compounds extracted from the plant *Tarphochlamys affinis* (Griff.) significantly caused the inhibition of the production of HBeAg and HBsAg. Huang et al. (2014) exhibited that PHAP (p-hydroxy acetophenone) extracted from the plant *A. morrisonensis,* effectively inhibited the replication of HBV-DNA. Zhao et al. (2015) discovered that PHAP and its derivatives exhibited activities against the viral DNA. A sequence of derivatives was obtained after the structural changes of p-HAP and its derivatives, amidst them, p-HAP derivative 2f demonstrated the most effective inhibition of the HBV-DNA replication (SI = 160:3, IC50 = 5:8 μM). The relationships between the primary structure and its activity indicated that the substituted cinnamic acids and the conjugated derivatives of p-HAP glycoside increased their actions against the replication of HBV-DNA.

### Artemisinin

Artemisinin is a plant-based product that is extracted from *Artemisia annua* and is a widely known antimalarial agent. It has been reported that Artemisinin also exhibits anti-HBV activities. Demonstrated that Artesunate, which is the semisynthetic derivative of Artemisinin demonstrated more effective activities by decreasing the amount of HBV-DNA at IC_50_ of 0.5 μmol/L and led to the inhibition of the production of HBsAg at IC_50_ value of 2.3 μmol/L. The values were not observed to be better compared to Lamivudine (IC_50_ of 0.3 μmol/L and 0.3 μmol/L), although a combination of both compounds produced a significantly effective result. As Lamivudine was subjected to drug resistance, thus, this combination effectively reduced the emergence of drug resistance against Lamivudine. Also, Artemisinin and artesunate do not cause serious side effects, regarding their anti-HBV effects.

### 2-Arylbenzofuran derivatives

They are obtained from MCR or Mori cortex radicis and exhibited anti-HCV effects in a system of HCV replicon (Lee et al., 2007). They also demonstrate anti-oxidative properties. The assay of NS3 helicase revealed that the two compounds demonstrated effective inhibitory effects (IC_50_ of 42.9 and 27.0 μmol/L). The NS3 viral helicase unwinds the duplexes of RNA × DNA as well as RNA × RNA, hence, is significant for viral replication. Therefore, targeting the NS3 helicase enzyme is ideally considered, and the by-products of this enzyme can be used for the development of effective inhibitors of helicase in the future (Lee et al., 2007). Dai et al. (2001) demonstrated that mellenin, a fungus-based compound, extracted from *Aspergillus ochraceus*, demonstrated anti-Hepatitis C virus protease activity with 35 μmol/L, as an IC_50_ value. Another study by Hu et al. (2007) included the screening of numerous compounds of pseudo guaianolides derived from *Parthenium hispidum*. The results revealed antiviral effects in a HCV subgenomic replicon system as three out of all the compounds caused inhibition up to around 90%, at a concentration of 2 μmol/L. Also, the other compounds demonstrated inhibition up to 50% without any cytotoxicity, thus, indicating further research for their higher potentials.

### Blueberry proanthocyanidins

According to the USDA database, every 100 g of the edible portion of blueberries contains about 88–261 mg of proanthocyanidin. They are structurally similar to polyphenols like flavonoids and anthocyanins (Huang et al., 2012). Proanthocyanidins exhibit anti-oxidative, anti-tumor, and anti-inflammatory effects (Yang et al., 2014). Blueberry as well as polyphenols demonstrate some important biological properties as antibacterial, neuroprotective, antiviral, anticarcinogenic, and cardioprotective agent (Joshi et al., 2016). Additionally, Takeshita et al. (2009) demonstrated that the fraction of methanol extract from blueberry leaves (0.112–2200 lg/ml) led to the reduction in the activities of subgenomic HCV in a replicon cell system of HCV after 72 h at a temperature of 37°C. Joshi et al. (2016) reported that blueberry juice and its proanthocyanidins (B type) exhibited anti-viral effects against HAV and are also effective against human norovirus. The study also showed that the HAV titers in the suspension decreased significantly to undetectable levels by proanthocyanidins at concentrations of 2 as well as 5 mg/ml in half an hour and by 1 mg/ml proanthocyanidins after 3 h. Furthermore, it was observed that within 24 h, blueberry juice (37°C and pH 2.8) significantly reduced HAV level (2 log PFU/ml). The blueberry juice and isolated proanthocyanidins were evaluated for their activity against HAV adsorption and replication in FRhK4 cells, after being pre- and post-infected with the HAV HM175 strain. The blueberry juice and isolated proanthocyanidins were significantly able to decrease the HAV level in the pre-infected cells, however, in the post-infected cells, there was no inhibition of the viral replication (Joshi et al., 2016).

### Cyclophilins

Cyclosporin A (CsA) exhibits immunosuppressive activity. CsA is a significantly effective anti-HCV compound based on the screening of cell cultures for anti-HCV products. It is a fungus-based compound that is produced by *Tolypocladium inflatum* Gams and exhibited significant biological applications in organ transplantation and immunosuppressive effects (Doutre, 2002). CyPB (cyclophilin PB), stimulates the anti-viral effects of CsA against HCV (Watashi and Shimotohno, 2007) and is primarily found in ER membrane’s cytoplasm. It is exactly similar to the NS5B polymerase of HCV. Additionally, both of these compounds establish complexes with HCV-RNA. Watashi et al. (2005) demonstrated that cyclophilin PB enhanced the RNA binding *via* NS5B, and the reduced levels of CyPB resulted in the lack of viral replication (Watashi et al., 2005). Further, the complex of NS5B and CyPB was disrupted by CsA, as a result of which, the replication of the HCV genome was reduced. As CsA exhibited immunosuppressive effects, therefore, it is not advisable for the treatment of HCV infections. However, NIM811 (another derivative having one substituted amino acid), demonstrates two-fold effective binding affinity to CyPB and lacks immunosuppressive activity. Five out of eight patients, who’d undergone liver transplantation and also suffered from HCV recurrence, did not respond to the standard HCV therapy, but the effective results of cyclosporin gave hope due to the reduced HCV-RNA below the level of detection (Watashi and Shimotohno, 2007).

### Ellagic acid

Ellagic acid, is a flavonoid product obtained from *Phyllanthus urinaria* and it demonstrates anti-oxidative, anti-inflammatory, and neuroprotective activities. It also demonstrated an interesting anti-HBV effect. Shin et al. (2005) exhibited that it hindered the secretion of HBeAg in cell culture with 0.07 μg/mL as an IC_50_ value, but it did not cause the inhibition of production of HBsAg, polymerase activity, and HBV replication. Another report by Kang et al. (2006) illustrated an experiment on the HBeAg-producing transgenic mice and showed that they exhibited significant effective tolerance towards HBeAg. As a result, there were reduced levels of CTL (cytotoxic T-lymphocyte) responses, minimal levels of cytokines production, and no secretion of antibodies to the antigen. But, when the mice were fed with ellagic acid, there was an inhibition of this immune tolerance and thus it was concluded that ellagic acid is considered an effective agent to overcome this essential mechanism against the chronic infection of HBV.

### Enediynes

Enediynes derived from the plant *A. capillaris* (Yin-Chen) is used as a therapeutic agent for hepatitis predominantly in China (Liu et al., 2019). They also demonstrate anti-tumor actions. Subsequently, Geng et al. (2015) exhibited that two glucopyranoside derivatives of enediynes caused effective inhibition of HBV DNA replication and the production of HBsAg along with HBeAg. These compounds exhibited inhibitory actions against the HBV-DNA replication with SI values equal to 17.1 and 23.6, as well as IC_50_ levels of 0:0127 ± 0:05 and 0:077 ± 0:04 mM. Also, a specific pair of isomers of enediynes demonstrated inhibitory activities of the production of HBsAg at IC_50_ of 0:887 ± 0:20 mM (SI = 2.3) and 0:797 ± 0:23 mM (SI = 2:1). This study even showed that one of the compounds exhibited most effective inhibitory actions against the replication of the viral DNA with SI = 23:6 and IC_50_ of 0:077 ± 0:04 mM, while the similar derivative with -(2′-O-caffeoyl), exhibited slightly reduced actions against HBV-DNA replication with SI = 17:1 and IC_50_ level of 0:127 ± 0:05 mM. Another study by Geng et al., 2018, extracted fourteen compounds from *A. capillaris* which were essayed for their structure-activity relationship, and their anti-HBV properties were summed up on the basis of their biological actions. Out of these, two of the compounds effectively caused the inhibitory actions against of the productions of HBeAg, HBsAg and HBV DNA replication at IC_50_ values of 48.7 (SI > 20:5), 197.2 (SI > 5:1), and 9.8 (SI > 102) μM.

### Essential oils

Essential oils (EO) are plant-based aromatic oils that are extracted from roots, grass, fruit, branches, flowers, bark, leaves, buds, wood, and seeds. Some EOs derived from sweet orange, rosemary cineole, lemon, and grapefruit (common names of *Citrus sinensis*, *Rosmarinus officinalis, Citrus limon,* and *Citrus paradisi,* respectively) exhibit anti-HAV effects (Battistini et al., 2019). Essential oils like sesquiterpenes, hydrocarbons, and limonene, which are produced by the genus Citrus, are 85%–99% volatile, with their oxygenated agents like esters, ketones, aldehydes (citral), alcohols (linalool) and acids (Fisher and Phillips, 2008). EOs exhibit antimicrobial, anti-cancer, anti-fungal, and anti-spasmodic activities. Battistini et al. (2019) conducted an experiment in which the Frp3 cells were inoculated with ATCC or HM-175 hepatitis A strain and then treated with rosemary cineole essential oil, after 30 min of incubation at room temperature. It was observed that the rosemary cineole essential oil led to an effective decrease of cell infectivity followed by lemon and grapefruit essential oils. Apart from this, it is essential to estimate the least time taken by essential oils to cause maximum effectiveness against the viral loads in food products in order to make people more aware about food safety (Battistini et al., 2019).

### Flavonoids

Flavonoids are plant-based products that exhibit numerous clinical functions, it acts as an anti-bacterial anticancer, and anti-inflammatory. It demonstrates a major function in the protection of the liver, like, Silymarin is an effective medicine that is developed for protecting the liver (Yi, 2012). Wang et al. (2013) exhibited that flavonoids are effective against HBV, wherein Luteolin caused the inhibition of the production HBsAg as well as HBeAg on the cells of HepG 2.2.15 *in vitro*, at IC_50_ value of 0.02 mM. Another plant-based flavonoid, Isovitexin, derived from *S. yunnanensis* also exhibited effective anti-HBV properties. Cao et al. (2013a) showed how it has not just prevented the HBsAg and HBeAg secretion (at IC_50_ levels of 0.04, less than 0.03, and 0.23 mM), and also inhibited the replication of HBV- DNA (at IC_50_ of 0.09, less than 0.01 as well as 0.05 mM). Cao et al. (2015) reported that Isoorientin (derived from *S. mussotie*) possessed anti-HBV functions in opposition to the production of HBeAg and HBsAg at IC_50_ of 1.12 and 0.79 mM, and the viral replication at IC_50_ of 0.02 mM. Another study by Xiao. (2018) proved the anti-HBV effects demonstrated by Epimedium Hyde II (a Chinese herbal compound). It also prevented the HBV-DNA replication and the activities of HBeAg and HBsAg in the HBV-replicated serum of the C57BL/6 mice. Additionally, various researchers discovered that isopongachromene and glabaarachalcone, which are plant-based compounds and are extracted from *P. pinnata* can be linked to the viral DNA polymerase protein target (Mathayan et al., 2019).

### Ginsenosides

Ginseng which is also known as *Panax ginseng* Meyer is popularly used in Korea and China as a medicinal herb for over 5000 years (Yun, 2001). They contain numerous bioactive compounds like peptides, polysaccharides, ginsenosides, fatty acids, phytosterols, poly acetylenic acids, and polyacetylenes. There are various studies on the biological activities of Ginseng like they are used as an antifungal, anti-stress, anti-inflammatory, anti-bacterial, anti-carcinogenic, antiviral, and anti-oxidant agent (Lee et al., 2011). Its accumulation is primarily reported in its plant’s roots and traditionally, isolation of the same usually takes an extended period of time (Luthra et al., 2021). Lee DY. et al (2013) exhibited that the Korean red ginseng (or KRG), as well as purified ginsenosides (Rg1 and Rb1), were used at different concentrations for the pre-treatment and co-treatment on FRhK-4 cells, after the inoculation of Hepatitis-A virus ATCC strain on the cell line. The outcomes demonstrated that both of the above-mentioned compounds effectively reduced the HAV levels. This research also exhibited that the KRG compound exhibited cytotoxicity exceeding 10 μg/ml concentration, but ginsenosides did not demonstrate any cytotoxicity up to the concentration of 40 μg/ml. Furthermore, even though KRG and the purified ginsenosides were used for the co-treatment of the cell lines, they effectively decreased the viral concentration and the pretreated cells exhibited significant anti-HAV effects. Therefore, pretreatment with ginseng significantly prevents HAV infection.

### Green tea catechins

Green tea catechins (GTCs) are natural and herbal compounds that are highly beneficial to human health. As the name suggests, they are the components of *Camellia sinensis,* of the family *Theaceae*. One of the most studied and abundant catechins is EGCG or (−)-epigallocatechin 3-gallate (EGCG), while others include EC or epicatechin, (−)-epigallocatechin (EGC) and (−)-epicatechin gallate. They comprise anti-cancerous, anti-oxidative, anti-infectious, and anti-inflammatory functions, according to *in vitro* as well as *in vivo* examinations (Cao et al., 2016). Ye et al. (2009) analyzed the anti-HIV actions of tea catechin mixtures on the production of Hepatitis-B-antigen and the production of DNA in a stable cell line of HBV-transfected HepG2. Other catechins and EGCG inhibits the production of the HBeAg as well as HBV DNA at a specific dosage with IC_50_ of 7.34 µg/ml and 2.54 µg/ml. He et al. (2011) showed that when HepG2.117 cells were made to grow when EGCG, HBeAg expression was suppressed, undisturbing the HBsAg expression. Chen C. et al (2012) proved the reduction of HCV infection *via* EGCG in the cells of Huh7.5.1 cells, with the help of the JFH1 strain of hepatitis C genotype 2a which produced the contagious viruses in the cell culture. This treatment for HCV with EGCG at EC_50_ was around 17.9 µM.

### Iminosugars

The potential ER (or endoplasmic reticulum) inhibitors α-glucosidases are basically the by-products of DNJ or deoxynojirimycin iminosugars. Such iminosugars are naturally present, for instance in silkworms or *Bombyx mori* (Jacob et al., 2007). They are potent immunomodulators and demonstrate anti-microbial properties. Iminosugars significantly reduce the virality of infectious viral particles. This suppression might be sourced by the incorporated envelope proteins (misfolded) in the secreted particles (Chapel et al., 2007). The derivatives of iminosugars have been proven to show antiviral effects against infections caused due to HBV as well as HCV. A product namely, 1-DNJ, extracted from the plant of *Morus alba* L, suppressed the hepatitis B viral particle secretion in a dose-dependent way (Jacob et al., 2007). Steinmann et al. (2007) reported that compounds having a long alkyl side chain significant for inhibitory effects on p7 (an ion channel of HCV) as well as a DNJ head group, lead to an advantage for susceptibility to resistance. Misumi et al. (2021) showed that Miglustat was used for treating lipid storage illnesses in humans, as well as UV-4 inhibited the replication of HAV in cell culture, at IC_50_ 32.13 μM as well as 8.05 μM, respectively *via* blockage of ganglioside synthesis (crucial for the HAV cell entry).

### Japanese rice-koji miso extracts

Koji (or *Aspergillus oryzae*) has been predominantly used by the Japanese for the fermentation of various food items like rice, soybean, grains, and potatoes. Miso is obtained as a by-product when Japanese rice is fermented by Koji. It is used as a seasoning in the preparation of Miso soup (Win et al., 2018). It exhibits antioxidant and anti-aging effects (Lee MH. et al., 2013). Jiang et al. (2011) demonstrated that Miso enhanced the effects of GRP78- also known as glucose-controlled protein 78, which is basically a heat-shock protein that resulted in the suppression of Ultraviolet C mutagenesis. Some researchers also demonstrated that the expression of GRP78 retarded the HAV replication (Win et al., 2018). Therefore, GRP78 acts against HAV infection as an effective antiviral agent (Jiang et al., 2017). Another report showed how Win et al. (2018) conducted a post-infection assay demonstrating that the Miso extract synergistically functioned as an antiviral against HAV infection by partially enhancing the effect of GRP78. It even stimulated the effects of GRP78 in PXB cells and Huh 7 (human hepatocytes) and suppressed the replication of HAV (Win et al., 2018). As a result, the Miso extract has been used as an important dietary product for controlling HAV infection.

### Lactoferrin

Lactoferrin is derived from cattle as well as camel milk and has been reported as a combinational therapy along with conventional hepatitis C drugs. It also demonstrates antimicrobial, immunomodulatory, and, anti-cancer activities. Many trials have confirmed the effective functions of camel milk-derived lactoferrin, regarding its therapeutic value against hepatitis (Adinolfi et al., 2001; Redwan and Tabll, 2007). It was mentioned in a study that lactoferrin derived from camel milk inhibited hepatitis C genotype 4 *via* the prevention of virus from the entering cells (Gader and Alhaider, 2016). The compound also showed antiviral, antifungal as well as antiparasitic activities, toward a wide spectrum of species (Jenssen and Hancock, 2009). EL-Fakharany et al. (2013), demonstrated significant activities of lactoferrin against hepatitis virus, isolated from camel milk was reported on the PBMCs or peripheral blood mononuclear white blood cells as well as HepG2 or human hepatoma infected cells having HCV. Redwan et al. (2014) reported the activities of lactoferrin on the Huh-7 cell line in a cell culture medium that was inoculated with HCV and further noted the dismantling of viral peptides and inhibition of the virus’s growth.

### Lignans

They usually extracted from plants. One of the primary groups of phytoestrogen, they play a role as antioxidants (Wohlfarth and Efferth, 2009). For instance, Honokiol is a lignan isolated from leaves, barks, and cones of *Magnolia officinalis*. Lan et al. (2012) assessed the effects of honokiol HCV infection; its entry, replications as well as translation, in the cell line Huh-7 using HCVcc, HCVpp as well as subgenomic replicons. The results showed that it strongly reduced the HCVcc infection (at EC_50_ of around 1.2 μg/ml, with respect to 4.5 μM, and EC_90_ of 6.5 μg/ml) at non-toxicity (median lethal dose = 35 μg/ml). Wu et al. (2012) proved the anti-HCV effects of another lignan namely, 3-hydroxy caruilignan C (or 3-HCL-C) which was isolated from the plant *Swietenia macrophylla* (stems)*.* The results also included that 3-HCL-C decreased NS3 proteins and the levels of HCV-RNA at EC_50_ of around 10.5 μg/ml (37.5 μM). Apart from this, 3-HCL-C is hindered with the replication of HCV as well, *via* induction of IFN-induced response element transcription as well as IFN-dependent anti-viral gene expression. Hence, 3-HCL-C is a potent adjuvant for the therapy of HCV.

### 4-Phenylcoumarin derivatives

Coumarin is a plant-based natural product that was first derived from *Dipteryx odoranta* and tonka beans. It is also known as Coumarou and there are numerous natural coumarins that are isolated from plants, fungi, bacteria, and chemical synthesis (Kassem et al., 2019). Coumarin along with its derivatives is used to synthesize antiviral agents (Garro and Pungitore, 2015). (2H-chromen-2-ones) are known to be superior bioactive agents for the synthesis of novel agents which possess high specificity and affinity to numerous molecular targets (Batran et al., 2018). Coumarin derivatives exhibit antioxidant, anti-inflammatory, neuroprotective and anti-cancer effects. Recently, it was reported that various derivatives of Coumarin exhibit anti-HAV activities (Kassem et al., 2019). Like picornaviruses, the Hepatitis-A virus genome encodes HAV 3C pro, also known as HAV three protease (an essential processing protease that is responsible to enhance viral replication by transcription, translation, and nucleo-cytoplasmic trafficking). 4-Phenylcoumarin-based compounds, which are recently modified antiviral compounds, target the 3C proteases and inhibit them (Kassem et al., 2019). There are various derivatives that demonstrate anti-HAV activity, which has been reported to exhibit the strongest virucidal activity and also inhibit the adsorption and replication of HAV, therefore, it possesses effective virustatic properties.

### Phenylalanine dipeptides

Dipeptide derivatives exhibit anti-inflammatory and antimalarial effects. A study by Yang et al. (2014) was conducted which extracted and altered the Matijin-Su (phenylalanine dipeptide) with anti-HBV actions from *Dichondra repens.* Forst, as well as four by-products, were tested with effective anti-HBV properties *in vitro*.

Another report by Meng et al. (2018) examined the twenty species of natural marine small molecules *via* the cells of HepG 2.2.15; three types of agents namely, 4-hydroxy proline-phenylalanine, glycine-L-proline, and L-2-hydroxy proline-phenylalanine demonstrated effects against the HBV by the hindrance of HBV-DNA, HBeAg and HBsAg. Wang et al. (2019) showed that N-acetyl phenylalanine demonstrated inhibitory effects on HBsAg as well as HBeAg at IC_50_ of 55.5 and 69.5 μg/ml, respectively. Kuang et al., 2019, utilized Matijin-Su (MTS) as a primary compound; and synthesized a novel derivative of MTS which demonstrated anti-HBV effects. Further, a series of derivatives of MTS were synthesized with Matijin-su as the primary compound, *via* incorporating chlorine or fluorine substitution, as well as the acquired derivatives of MTS, and were evaluated for anti-HBV actions *in vitro*. These outcomes demonstrate that the extracted compounds exhibited anti-HBV effects at IC_50_ values of 10.53, 12.61 12.61 mol/L.

### Phenylpropanoids

Phenylpropanoids are usually derived by plants from tyrosine and phenylalanine amino acids and comprise a broad spectrum of biological activities like antioxidation, antitumor, liver protection as well as antivirus. Chen H. et al. (2012) demonstrated that the extraction of a sequence of phenylpropanoids from the roots or core materials or bark of *S. asper* caused anti-HBV effects. Compounds like Magnatriol B displayed mild anti-HBV activity and inhibited both HBsAg as well as HBeAg secretions with lower cytotoxicities. Honokiol, on the other hand, showed strong inhibition on HBeAg as well as HBsAg with IC_50_ of 4.74 μM (SI = 14:22) and 3.14 μM (SI = 21:47), respectively. Isomagnolol and isocarpine, isolated from the roots or bark of the plant *S. asper* displayed prominent anti-HBV effects *via* the cell assay of HepG 2.2.15 and reduced the HBsAg production at IC_50_ values 10.34 μM as well as 3.67 μM. Also, for inhibition of HBeAg production, the IC_50_ was around 8.83 μM as well as 14.67 μM, at non-toxic levels. Another compound, Coumarin lignan, was derived from the plant of *Kadsura heteroclita* (stems), and caused inhibition of HBsAg as well as HBeAg production at a concentration of 25 μg/ml. Niranthin, derived from *Phyllanthus niruri*, also suppressed the HBsAg as well as HBeAg secretion at specific dosages, with IC_50_ of 16.5 μM as well as 25.1 μM, respectively.

### Polyphenols

Polyphenols demonstrate antioxidant, neuroprotective and anti-inflammatory effects. Many polyphenols exhibit anti-HCV activities. Nobiletin or 3′,4′,5,6,7,8-hexamethoxyflavone derived from the extract of *Citrus unshiu* was responsible for demonstrating anti-HCV effects (Suzuki et al., 2005). Hegde et al. (2003) proved that nobiletin showed activity against hepatitis C infection at 10 μg/ml in the MOLT-4 cells. Another study involved the isolation and characterization of two novel compounds-oligophenolic in nature, namely SCH 644343 as well as SCH 644342 from the plant of *Stylogne cauliflora* wherein they demonstrated inhibitory actions against HCV NS3 protease activity *in vitro*, with IC_50_ values of 0.3 μM as well as 0.8 μM (Hegde et al., 2003). Duan et al. (2004) recognized three polyphenol compounds from ethyl acetate fraction of Galla Chinese which is traditional Chinese medicine and showed that they inhibited NS3 protease *in vitro* at IC_50_ values of 0.75, 1.60, and 1.89 μM.

Zuo et al. (2005) reported one of the compounds inhibited the NS3 protease, derived from *Saxifraga melanocentra* Franch. The results showed that the IC_50_ value was 0.68 μM as well as the compound was safe till 6 mg/ml (or 6.4 mM) on the COS cells. Li Y. et al. (2012) characterized four polyphenolic compounds isolated from *Excoecaria agallocha* L. which inhibited NS3 protease *in vitro*. Out of these, two compounds namely, excoecariphenol D as well as corilagin displayed a prominent inhibitory effect in the replicon assay, with IC_50_ of 12.6 as well as 13.5 μM.

### Protamine, taxifolin and atropine

Taxifolin which is also known as Dihydroquercetin is a plant based product that is obtained from onions, grapes, citrus fruits, and olive oil. It takes a major part in the prevention of Alzheimer’s disease and was known for its effective pharmacological actions, which included, anti-diabetic, antitumor, antioxidative, hepatoprotective, cardioprotective as well as neuroprotective effects.

Protamine is an animal-based compound that is derived from fish milt. It is a cationic peptide and possesses numerous properties. It was used in the form of an antibacterial agent in food items and apart from that, it was used as a heparin antagonist and as an injectable-insulin carrier (Gill et al., 2006). Atropine is also a plant-based product that is derived from *Belladonna*. It is an anticholinergic agent (muscarinic receptor antagonist) which is administered to regulate the contractions as well as dilations of muscles in order to maintain the blood flow in cells (Behcet, 2014). Earlier, a study was conducted that demonstrated the significant inhibitory actions of Taxifolin, Protamine, and Atropine against the replication of HAV in the cells of PLC/PRF/5. Atropine resulted in a dose-dependent decrease in HAV infectivity. Taxifolin, Protamine, and Atropine, at a maximum concentration of 59, 50 as well as 50 μg/ml, respectively, decreased the HAV titer.

### Resveratrol

Resveratrol, which is also known as 3,5,40 -trihydroxystilbene, is a naturally derived phytoalexin. It is commonly found in plants like grapes, cranberries, peanuts, etc. It exhibits numerous biological activities as it is administered as a vasoprotective, chemopreventive, anti-inflammatory, and antioxidant compound (Ungvari et al., 2007). It has been studied that Resveratol causes inhibition of liver steatosis (ethanol-induced) in rats (Kasdallah-Grissa et al., 2006). Bujanda et al. (2008) reported that in rats with fatty liver infection (non-alcoholic) and found that it inhibited *de novo* lipogenesis of adipocytes, adipogenic differentiation, and reduced hepatic steatosis. Another report by Jiang et al. (2012) showed that Resveratol exhibited effective activity against HCV core protein-stimulated hepatic steatosis by enhancing the PPAR-a levels, which was inhibited through the HCV core protein, *in vivo* and *in vitro*.

### Silverstrol

It is extracted from *Aglaia foveolata* (Kim et al., 2007). Silverstrol exhibits anti-leukemia effects. It causes effective inhibition of the DEAD-box RNA helicase eIF4A (Bordeleau et al., 2008), which is a member of the eIF4F complex that is responsible for the cap-dependent initiation of eukaryotic translation (Silvera et al., 2010). Todt et al. (2018) exhibited that silvestrol has inhibitory actions against the HEV genotypes replication at a specific dosage. Zhou et al. (2015) examined the actions of the eIF4F complex in the HEV replication as well as reported that with respect to the silverstrol’s activity, effective HEV replication required the machinery eIF4A, eIF4G as well as eIF4E. Further, the study also reported that programmed cell death 4 (or PDCD4) as well as eIF4E-binding protein 1 (or 4E-BP1)- the negative regulatory factors with respect to the complex, displayed anti-HEV effects, thus proving the necessity of both eIF4A and eIF4E in the replication of HEV. As a result, silvestrol targeted these mRNA translation host factors for its antiviral effects. Another study by Glitscher et al. (2018) identified silvestrol as an efficient inhibitor of the viral particle release of HEV. The results showed a highly decreased HEV capsid protein translation as well as control of the viral RNA inside the cytoplasm, in the absence of any prime cytotoxic effects.

### Terpenoids

Terpenoids are natural compounds with a basic structural unit as isoprene. They exhibit effective biological actions, which mainly comprise antivirals and anti-inflammatory actions (Zhang et al., 2018). Li L. Q. et al. (2012) extracted ursolic acid from the core of *S. asper.* Ursolic acid possesses effective anti-HBV properties by inhibition of HBeAg as well as HBsAg secretion, at IC_50_ 97.61 and 89.91 μM. Another triterpenoid, MH, is a plant-based compound and is extracted from *Vicia tenuifolia* Roth and it demonstrated inhibitory action on the production of HBeAg as well as HBsAg at a specific dosage (Huang et al., 2013). Zhou et al. (2013), isolated heptane terpenoids from the rhizomes and roots of the plant *Aster tataricus*- Andepishionol and Astartaricusones B, which caused inhibition of the production of HBeAg, at IC_50_ values of 18.6, 40.5 μM. It also inhibited the replication of HBV-DNA at IC_50_ of 2.7, 30.7 μM. Another report by Liu and Wu. (2013) proved that Diosgenin significantly led to the suppression of HBsAg along with HBeAg secretion at the rate of inhibition of 40%–50%. Zhao et al. (2014) showed that Pumila Side A and 7-Eudesma-4 (15)-ene-1β,6α-diol, extracted from *Artemisia capillaris,* demonstrated effective actions against the replication of HBV-DNA at IC_50_ of 19.70, 12.01 μM (high SI values equivalent to 105.5, 139.2). Moreover, Pumila Side A suppressed the production of HBsAg as well as HBeAg at IC_50_ of 15.02 μM (SI of 111:3) and 9.00 μM (SI of 185:9). Swertia Side is a plant-based product which not only revealed the most effective pursuit against replication of HBV-DNA at IC_50_ of 0.05 mM (SI of 29:1), but also acted against the production of HBsAg (IC_50_ equivalent to 0:79 mM) (Jie et al., 2015). In addition to this, Laurifolioside and Genkwanin, extracted from *Wikstroemia chamaedaphne* Meisn, demonstrated significant anti-HBV actions at IC_50_ of 46.5, 88.3 mg/ml. Further, 2-epi-laurifolioside, Wikstroelide W, laurifolioside, 2-epi-laurifolioside A, laurifolioside B, and 2-epi-laurifolioside B exhibited inhibitory activities against the replication viral DNA to a certain extent, within the range of levels from 0.39–6.25 mg/ml and the ratios of inhibition ranging between 2.0% and 33.0% (Zhang et al., 2017).

### Wogonin

Wogonin is a plant-based compound that is extracted from *Scutellaria radix* and is a known mono-flavonoid. This herb exhibited biological activities against hepatitis and inflammatory diseases and has been administered in Asia for over a thousand years (Wohlfarth and Efferth, 2009). Huang et al. (2000) exhibited antiviral activities against HBV of this compound by illustrating its activities against the HBsAg secretion in the cell suspension. Guo et al. (2007) noted that it led to the suppression of HBsAg and HBeAg secretions at IC_50_ equivalent to 4 μg/ml. Furthermore, the study showed that Hepatitis-B virus DNA was decreased at a dose-dependent way. The results were tested on a bunch of ducks infected with DHBV (or duck-hepatitis B virus), proving the decreased rates of DHBV-DNA and plasma HBsAg and thus an improvement in the functions of the liver after the histopathological evaluations. Other results were seen on the human HBV-transgenic mouse livers treated with Wogonin, which led to the reduction of HBsAg after the immunological staining.

### Xanthones

Xanthones demonstrate anti-inflammatory, anti-cancer, and antioxidant activities (Shagufta., 2016). There are various types/derivatives of Xanthones which exhibited effective inhibition of the replication of HBV-DNA between IC_50_ from 0.01 to 0.13 mM (Cao et al., 2013b). This report also noted that the compounds which possessed hydroxy groups (3 or more) like 1,5,8-trihydroxy- 3-methylxanthine, Norbellidifolin as well as 2-C-β-D-glucopyranosyl-1,3,7-trihydroxy xanthone demonstrated effective inhibitory action against HBV at IC_50_ of <0.62, 0.35, and 0.04 mM for HBeAg as well as 0.77, >0.98, and 0.21 mM for HBsAg. Apart from this, Norswertianolin, Neolancerin, and 1,8-Dihydroxy-3,5-dimethoxy xanthone were derived from *S. yunnanensis* and demonstrated effective anti-HBV activity. Amongst the three compounds, Neolancerin not only prevented the HBsAg as well as HBeAg secretions at IC_50_ of 0.21, 0.10, and 1.51 but also hindered the viral DNA replication at IC_50_ of 0.09 mM, less than 0.01 mM, and 0.05 mM (Cao et al., 2013a). Cao et al. (2015) showed that 1,5,8-Trihydroxy-3-methylxanthine exhibited the inhibition of replication of the viral DNA at IC_50_ of 0. 09 as well as 0.05 mmol·L-1 (SI = 10. 89) and demonstrated a significant action against the HBeAg secretions at IC_50_ of 0. 35 (SI of ≥2. 80). Qin et al. (2016) reported that a natural compound extracted from *Penicillium* sp. Effectively reduced the HBsAg secretions than 3TC (positive control) at a dose-dependent way. Tables 1, 2, 3.

**TABLE 1:** Natural products for hepatitis A treatment.

Natural product	Mechanism of action	Concentration	Result of action	Reference
Blueberry Proanthocyanidins	Interruption of binding of HAV and its entry into the cell	2–5 mg/ml at a temperature of 37°C for half-hour	Decrease in HAV levels to undetectable measure in the medium	Joshi et al. (2016)
Essential Oil (or EO) derived from rosemary cineole, lemon and grapefruit	-	0.05% of rosemary cineole EO, 0.5% of lemon EO, and 0.1% of grapefruit EO	Decrease in cell infectivity (rosemary cineole > grapefruit > lemon)	Battistini et al. (2019)
Grape seed extract	Interruption of HAV binding to cell membrane receptors; prevention of adsorption	2 mg/ml at 37°C for 6 h	Suppression of HAV titer to undetectable levels	Joshi et al. (2015)
Green tea essence/extract	Binds to them and interferes with the viral attachment with the cell membrane receptors	5 mg/ml at 37°C for 2 h with pH 7.2	Total HAV inactivation in the suspension culture	Steinmann et al. (2013)
Japanese rice-koji miso extracts	Reduction in replication of HAV *via* improvement of GRP78 levels in human hepatocytes	-	Reduction in HAV replication	Win et al. (2018)
KRG or Korean Red Ginseng Extract and Ginsenosides	Activating the RNaseL pathway and boosting the cytokine production	5–10 μg/ml at a temperature of 37°C for 24 h	Suppression of HAV in a dose-dependent manner	Lee et al. (2013a)
4-phenylcoumarin derivatives	Interruption of adsorption of HAV on cell surface	10 μl at a temperature 37°C	Inhibition of HAV’s 3C protease activity	Kassem et al. (2019)

**TABLE 2:** Natural products for hepatitis B treatment.

Natural compound	Plant source	Target	Concentration	Result of action	Reference
Aloe-emodin	*Aloe vera*	CYP3A4	10 μg/ml	Reduction in HBeAg expression in the cells of HepG2.2.15	Parvez et al. (2019)
Aloin B	*Aloe vera*	HBV-DNA polymerase	50 μg/ml	Suppression of HBV antigen	Parvez et al. (2019)
Diterpenoids	*W. chamaedaphne*	HBsAg	0.016 μg/ml	Exhibited strong anti-HBV activity	Zhang et al. (2017)
Esculetin	*M. fortunei*	HBV DNA, HBsAg and HBeAg	-	Inhibition of antigens of HBV as well as HBV DNA expression *in vitro* along with HBV replication	Huang et al. (2019)
Glabaarachalcone	*P. pinnata*	HBV-DNA	5 mg/ml	Inhibition of virus binding	Mathayan et al. (2019)
Hypericin	-	HBV DNA, HBsAg and HBeAg	-	Notable inhibition of HBV DNA, HBsAg, and HBeAg	-
Rosmarinic acid	-	ε-Pol binding	-	Inhibition of HBV replication	Tsukamoto et al. (2018)
Rubiadin	*P. connata*	HBeAg as well as HBsAg	20 μg/ml for 10 min	Reduction of HepG2.2.15 cells and reduction of HBx expression levels	Peng et al. (1970)

**TABLE 3:** Natural products for Hepatitis C treatment.

Natural compounds	Source	Target	IC_50_ (*in vitro*)	Result of action	Reference
EGCG	*Camellia sinensis*	HCV virion	5–21 μM	Inhibition of cell-free virus transmission, as well as HCV cell-to-cell, spread, leading to undetectable infection levels	Calland et al. (2012)
Honokiol	*Magnolia grandiflora*	NS5B polymerase, NS3 protease and NS5A	4.5 μM	Active against HCVcc infection at non-toxic concentration; reduction of HCV replication at a dose-dependent way in both 1b and 2a subgenomic replicons	Lan et al. (2012)
3-hydroxy caruilignan C	*S. macrophylla*	HCV RNA and NS3 protease	37.5 μM	Expressed anti-viral activities against HCV at both protein as well as RNA levels (in safe concentrations)	Wu et al. (2012)
Ladanein-BJ486K	*M. peregrinum*	HCV RNA	2.5–10 μM	Efficient against all major HCV genotypes and inhibited HCV infection	Haid et al. (2012)
Luteolin–Apigenin	-	NS5B polymerase	1.1–7.9 μM	Inhibition of HCV activity and against the NS5B enzyme	Liu et al. (2012)
Naringenin	Grapefruit	HCV life cycle	109 μM	Non-toxic inhibition of HCV	Goldwasser et al. (2011)
Quercetin	*Embelia ribes*	NS3 protease	-	Inhibitory effect on HCV NS3 protease, reduction of viral production *via* suppression of NS3 required for HCV replication	Bachmetov et al. (2012)
Silymarin or Silibinin	*Silybum marianum*	NS5B polymerase	40–100 μM	Inhibition of HCV replicon as well as JFH1 replication in cell culture, suppression of HCV RNA-dependent RNA polymerase activity	Ahmed-Belkacem et al. (2010)

### Future perspectives

In recent times, many studies have explored the potential of natural compounds as an emerging treatment option for hepatitis. Even though vaccines have been developed for mitigating the spread of viral infection for a considerable time period, there is an urgent requirement for developing efficient anti-hepatitis adjunctive, therapeutic as well as prophylactic agents. Moreover, naturally derived compounds with beneficial potential against hepatitis have been investigated; some of which have even displayed prominent potential for control of hepatitis. Furthermore, the studies should be focused on imitating the characteristics of the hepatitis virus *in vivo* rather than *in vitro* in order to properly demonstrate the foundation for the applications of such natural compounds in a clinical setup. There is a requirement for the development of more suitable animal models which would display more or less the same clinical demonstrations as seen in humans suffering from hepatitis, for more precise elucidation as well as the correlation of results from pre-clinical trials including natural agents therapy. Also, more drug combinations with different targets, may have better therapeutic intevention. As a result, natural products should be included in the development of newer therapies, since they show promising and potent effects and may hence, replace the current standard and aggressive therapies.

## Conclusion

In recent times, because of the after-effects plus the appearance of medicinal residues in chemical drugs in hepatitis treatment, more attention is being paid to naturally derived medicines. As a result, it is extremely important to study as well as address efficient natural compounds for curing hepatitis and its types, as also their action mechanisms. These compounds are a viable source for the synthesis of novel drugs for the treatment of hepatitis. They range in different bioactivities which can be directly developed or administered as initial points for novel drug optimization. In addition to this, clinical trials have shown that bioactive products have the potential to treat hepatitis, mostly HBV and HCV infections. Therefore, this review can provide a strong foundation for bioactive compounds to be used for the treatment of hepatitis.

## Abbreviations

HAV, Hepatitis A virus; HBV, Hepatitis B virus; HCV, Hepatitis C virus; HDV, Hepatitis D virus; HEV, Hepatitis E virus; ALT, Alanine transaminase; AST, Aspartate transaminase; IFN, Interferons; PEG-IFN-α, Pegylated interferon alfa; EMA, European medicines agency; DHCH, Dehydrocheilanthifoline; PHAP, p-hydroxy acetophenone; PFU, Plaque forming unit; CTL, Cytotoxic T-lymphocyte; EO, Essential oils; GTC, Green tea catechins; EGCG, Epigallocatechin 3-gallate; PBMC, Peripheral blood mononuclear cells.
